# Examination of the Activity of Camel Milk Casein against Hepatitis C Virus (Genotype-4a) and Its Apoptotic Potential in Hepatoma and HeLa Cell Lines

**DOI:** 10.5812/kowsar.1735143X.722

**Published:** 2011-09-01

**Authors:** Osama Almahdy, Esmail M. EL-Fakharany, Ehab EL-Dabaa, Tzi Bun Ng, Elrashdy M. Redwan

**Affiliations:** 1 Antibody Laboratory, Protein Research Department, Genetic Engineering and Biotechnology Research Institute, Alexandria, Egypt; 2 Department of Biochemistry, Theodore Bilharz Institute, Cairo, Egypt; 3 School of Biomedical Sciences, Faculty of Medicine, the Chinese University of Hong Kong,Shatin,N.T., Hong Kong, China

**Keywords:** Camel, Casein, Hepatitis C virus, Blocking, Cell entry, Apoptosis

## Abstract

**Background:**

Hepatitis C is a global health concern that represents a major cause of liver disease and socioeconomic burden. Currently, there is no vaccine that protects against this infection or drug that treats it effectively. The current treatment for hepatitis C virus (HCV) infection does not produce a sustained virologic response. Therefore,discovery and identification of a new drug for HCV treatment is a high priority.Camel milk is a traditional medicine that could improve the control of HCV.

**Objectives:**

To assess the potential effect of casein purified from camel milk on HCV cellular infectivity in a tissue culture model.

**Materials and Methods:**

Casein was purified from defatted camel milk to electrophoretic homogeneity. PBMCs and HepG2 and HeLa cell lines were used. Three kinds of experiments  were conducted. HCV was directly interacted with casein and then mixed with different cell types, casein was incubated with the cells and then exposed to HCV, and the HCV pre-infected cells were treated with casein at different concentrations and time intervals. Non-infected cells were used to assess cytotoxicity and the apoptosis effect of casein.

**Results:**

Direct interaction of casein (with or without α-lactalbumin) with neither the virus nor the cells prevented HCV cell entry. However, casein with α-lactalbumin induced a cytotoxic effect in HepG2 and HeLa cell lines but not in human naïve leukocytes. At all concentrations tested, casein with α-lactalbumin could induce apoptosis in both infected and non-infected HepG2 cells.

**Conclusions:**

Camel milk casein (with or without α-lactalbumin) did not demonstrate any anti-HCV activity. However, the cellular apoptotic cascade was initiated in HepG2 and HeLa cells treated with casein (with α-lactalbumin) but not in naïve leukocytes.

## 1. Background 

Globally, over 170 million people are infected with the hepatitis C virus (HCV), and the devastating impact of this virus is further magnified by the high frequency of HCV persistence during infection, i.e. a chronic infection results in up to 85% of cases. HCV infection has become the most common cause of hepatocellular carcinoma and the primary reason for liver transplantations among adults in the Western world [1]. Egypt has the highest number (12%) of HCV-infected patients and consequently a larger portion of its population is at risk of infection [2][3]. In Egypt, The majority of HCV-infected patients are concentrated in villages and terminal urban and central cities where income rates range from low to very low. Interferon-α2a remains the drug-of-choice, despite its low efficacy and adverse side effects [4]. Combination therapy using pegylated IFN-α2a and ribavirin improves the response rate to around 60% with fewer side effects [4], but still the economical factors standard special at developing countries.

Camel’s milk is an important nutritional source in several world regions, and it is consumed fresh or curdled. Often, HCV-infected Egyptian patients consume large amounts of camel’s milk as an alternative and/or supportive medicine [5]. To explore the macromolecules in camel’s milk that may be responsible for its protective potential, we previously screened and analyzed the potential anti-HCV activity of 2 camel milk proteins: lactoferrin and amylase. Lactoferrin showed significant activity against HCV cell entry into both human naïve leukocytes and HepG2 cells, while amylase failed to exert any anti-viral activity in the same tissue culture system. These studies did not find any cytotoxic effect for camel milk lactoferrin or amylase on naïve leukocytes or HepG2 cells [5][6][7]. Both lactoferrin and amylase represent minor proteins in camel milk. However, camel casein accounts for 80% (v/v) of the whole protein inventory, which can be simply isolated from skimmed milk by lowering the pH.

## 2. Objectives 

The current investigation focused on exploring the anti-HCV potential activity of purified camel milk casein on both naïve leukocytes and HepG2 cells.

## 3. Materials and Methods

### 3.1. Processing of Camel Milk and Casein Purification

Milk from camels (Camelus dromedarius) was purchased from ALKHIR camel farm (Giza, Egypt) and transferred frozen to our laboratory. A solution of sodium azide (0.2%) containing 5 mM EDTA and 5 mM PMSF was added to the milk before defatting by centrifugation at 1000 × g for 30 min at 4 oC. The pH of skimmed milk was decreased to 4.6 with 1 M HCl to precipitate the casein. After centrifugation at 5000 × g for 15 min, the casein pellet was separated from the whey supernatant and washed 2 times with cold distilled water, then further purified by readjusting its pH twice to exclude α–lactalbumin, which yielded casein-free α-lactalbumin (casein without α-lactalbumin), and then lyophilized.

### 3.2. Protein and Endotoxin Determination

Protein content was determined by directly measuring the absorbance at 280 nm with the Bradford method [8], using bovine serum albumin as the standard protein. The endotoxin level in the casein was checked [9] to avoid its pyrogenic effects on the cell-culture system. All casein batches used were free of endotoxin (data not shown).

### 3.3. Sodium Dodecyl Sulfate-Polyacrylamide Gel Electrophoresis (SDS-PAGE)

To examine the homogeneity and molecular weight of purified casein, it was subjected to electrophoresis under reducing conditions with the use of a 12% SDS polyacrylamide gel. The gel was stained and destained according to previous standard protocols [10].

### 3.4. Infected Serum Samples

For all infection experiments, PCR-HCV positive serum samples of genotype 4a from Egyptian patient “A.R.” (after approval by the ethics committee) were used as previously described by Redwan and Tabll [5].

### 3.5. Cytotoxicity of Casein

The 3-(4, 5-dimethylthiazol-2-yl)-2, 5-diphenyltetrazolium bromide (MTT) assay is a simple non-radioactive colorimetric assay for measuring cell cytotoxicity, proliferation or viability [5][11][12] In brief, peripheral blood mononuclear cells (PBMCs) were isolated [5], about 104 PBMCs and HepG2 cells in 150 µL complete media (5% fetal calf serum, 2 mM l-glutamine, 1 mM sodium pyruvate, 100 µg/mL penicillin-streptomycin, and 50 µg/mL gentamycin, and 50 µM 2-mercaptoethanol) were plated per well in a 96-well plate. After incubation (37 oC, 5% CO2) overnight, the cells were treated with camel casein (2 mg/mL) for 4 and 8 days. The cells were washed 3 times with 200 µL PBS, and 200 µL MTT solution (0.5 mg/mL in PBS) was added to each well. After further incubation (37 oC, 5% CO2) for 3–4 h, the medium was discarded, and the wells were dried. Formazan crystals were resuspended in 200 µL dimethyl sulfoxide, followed by shaking for 5 min to thoroughly mix the formazan into the solvent. The optical density was read at 550 nm. The relative cell viability (%) compared to control wells containing cell culture medium without casein was calculated using the following formula: (A)test/(A)control× 100%.

### 3.6. Inhibitory Potential of the Casein on HCV

To examine the interaction of casein with human PBMCs (2.5 × 105) and HepG2 cells, 105 cells were plated in two 24-well microtiter plates. Casein (free or containing α-lactalbumin) was added to the leukocytes and HepG2 cells (in 50 mL of RPMI-1640-supplemented medium) at a final concentration of 2.0 mg/mL for each type of cell and incubated for 60 min at 37ºC. Free casein was removed by washing 3 times with 1 mL PBS. After adding 1 mL of 2% HCV-infected serum (8.3 × 106 copies/mL, genotype 4a) to each well, the cells were incubated for 90 min at 37ºC, then washed 3 times and cultured for 7 days at 37ºC. To examine the interaction of casein with HCV, 1 mL of infected serum and casein (at a final concentration of 2.0 mg/ mL) was pre-incubated in 50 mL of medium for 1 h at 4ºC. Then, the mixture of HCV and casein was added to leukocytes or HepG2 cells cultured as described above and then incubated for 90 min at 37 ºC. However, the treatability of casein was examined as follows. Pre-infected cells were exposed to purified casein with and without α-lactalbumin at concentrations of 0.25, 0.5, 0.75, 1.0, and 2.0 mg/mL after 4 and 8 days from infection, respectively. The negative control was infected cells cultured without treatment. The cells were washed 3 times with 1 mL of medium and further cultured for 7 days at 37 ºC, followed by total RNA extraction [5].

### 3.7. Intracellular HCV Immunostaining Assay

The intracellular HCV immunostaining assay was performed in a 24-well microtiter plate. Casein (2 mg/mL) was incubated with infected serum for 1 h at 4 ºC, and then the mixture of HCV and casein was added to cells cultured as described above and incubated for 90 min at 37 ºC. The cells were washed 3 times with 1 mL medium and further cultured for 7 days at 37 ºC. On day 8, blocking buffer (2% bovine serum albumin or gelatin in PBS) was added and left for 1 h at room temperature. Then, the cells were fixed with paraformaldehyde and permeabilized with 0.5% Triton-X100. Purified primary antibody [11][13] diluted to 1:2000 was then added, followed by incubation for 1 h at room temperature. Horseradish peroxidase-conjugated anti-human IgG (diluted 1:2000 with 2% bovine serum albumin or gelatin-PBS) were added to the cell monolayer and incubated for 1 h at room temperature. The reaction was developed with 3, 3′-diaminobenzidine (DAB)-peroxidase substrate and stopped after 10 min of incubation with distilled water.Positive and negative controls were included. The distinct colored foci were examined, and some positive and negative fields were pictured by using a phase contrast microscope Olympus 1X70 (Olympus optical, Tokyo, Japan).

### 3.8. Extraction and PCR of Genomic and Anti-genomic RNA Strands of HCV

Strands of HCV RNA were isolated from PBMCs and HepG2 cells as previously described [3][5].Reverse transcription-nested PCR was carried out as previously reported [5][7].Retrotranscription was performed in a 25 µL reaction mixture containing 20 U of AMV reverse transcriptase (Clontech) with either 400 ng of total RNA of HepG2 cells or PBMCs, 40 U of RNAsin (Clontech), a final concentration of 0.2 mmol/L of each dNTP (Promega, Madison), and 50 pmoL of the reverse primer 1CH: 5′-GGTGCACGGTCTACGAGACCTC-3′ (for the plus strand) or 50 pmol of the forward primer 2CH: 5′AACTACTGTCTTCACGCAGAA-3′ (for the minus strand). The reaction mixture was incubated at 42ºC for 60 min and denatured at 98 for 10 min. The highly conserved 5′-UTR sequences was amplified using 2 rounds of PCR with 2 pairs of nested primers. First-round amplification was performed in a 50µL reaction mixture containing 50 pmol each of 2CH forward primer and P2: 5′-TGCTCATGGTGCACGGTCTA-3′ (reverse primer), 0.2 mmol/L of each dNTP, 10 µL from RT reaction mixture as template and 2 U of Taq DNA polymerase (Promega) in a 1 × buffer supplied with the enzyme. The thermal cycling protocol was as follows: 1 min at 94ºC, 1 min at 55ºC, and 1 min at 72ºC for 30 cycles.The second-round amplification was done similar to the first-round, except for the use of the nested reverse primer D2: 5′ACTCGGCTAGCAG TCTCGCG-3′ and forward primer F2: 5′-GTGCAGCCTCCAGGACCC-3′ at 50 pmol each. A 174 bp fragment was identified in the positive samples.

### 3.9. Apoptosis Assessment

3.9.1. DNA Fragmentation Assay

Cells were harvested after 8 days of casein treatment,washed with cold PBS, and then the oligonucleosome length DNA fragments in the samples were detected by agarose gel electrophoresis [14]. In brief, the cells were suspended and centrifuged, then lysed in 5 mM Tris, containing 20 mM EDTA, 0.5% Triton-X100, pH 8.0 at 4oC overnight and centrifuged at 16000 × g for 20 min. DNA oligosomes in the supernatant were precipitated with ethanol overnight at –20oC, treated with proteinase K (50 µg/mL) and RNase (50 µg/mL), and then loaded on 1.8% agarose gel. The samples were electrophoresed at a constant voltage 100 V for 60 min. The gel was stained with ethidium bromide and then visualized and photographed on the UV transilluminator of a gel documentation system.

3.9.2. Acridine Orange/Ethidium Bromide (EB/AO) Staining Assay

The dye mix of EB/AO consisted of 100 µg/mL ethidium bromide and 100 µg/mL acridine orange in PBS. The treated cells (PBMCS and HepG2) were stained with EB/AO. The EB/AO dye mix (8 µL) was added to each well, and the cells were viewed and counted after 15 min with an Olympus 1X70 (Olympus, Japan) inverted microscope at 40x magnification with an excitation filter (480/30nm), a dichromatic mirror cut-on 505 nm LP. Pictures were taken with an Olympus microscope equipped with a digital camera. All tests were done in triplicate. A minimum of 100 cells were counted.

## 4. Results

### 4.1. Purification of Camel Casein

**Figure 1:**
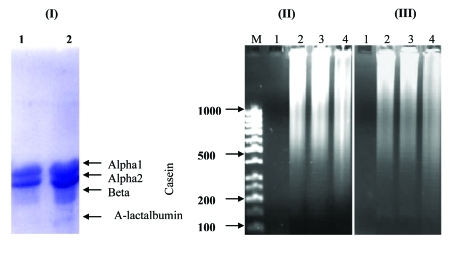
(A) 12% SDS-Urea PAGE of Casein Purified from Camel Milk. Four bands: 3 with molecular weights of 27.7, 24.9, 22.0 kDa, and α-lactalbumin with a molecular weight of 14.4 kDa. Lane 1 is repurified casein without α-lactalbumin, and lane 2 is purified casein with α-lactalbumin. (B) The agarose gel picture reveals the effect of camel casein on HepG2 and HeLa cells (II and III, respectively). The apoptotic DNA nucleosomes were run in 1.8% agarose gel after 8 days of casein (with α-lactalbumin) treatment. Lane 1 was the negative control (untreated cells). Lane 2 was the positive control (cells treated with the apoptosis inducer H2O2). Lane 3 was cells treated with 0.5 mg/mL casein, and lane 4 was cells treated with 2.0 mg/mL casein. DNA fragmentation was visualized with ethidium bromide as described in Materials and Methods.

Ten liters of Arabian camel milk was defatted. Casein was isolated from the acidified defatted milk, which yielded 4 and then 3 bands in SDS-PAGE after repurification. The molecular mass of camel casein was estimated to be 27.7, 24.9, and 22.0 kDa and that of α-lactalbumin was estimated to be 14.4 kDa (Figure 1).

### 4.2. Inhibition Potential of Camel Casein

**Figure 2:**
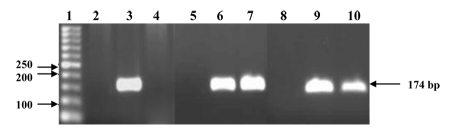
Camel Casein Activity against HCV. Lane 1 is the DNA ladder, lanes 2 and 5 are negative control samples for HepG2 cells, and lanes 3 and 6 are positive control samples for HepG2 cells. Casein with and without α-lactalbumin at a concentration of 2.0 mg/mL was tested for its ability to block HCV entry into HepG2 cells (lanes 4 and 7, respectively). Lanes 8 and 9 are the negative and positive control samples for PBMCs, respectively, and casein at a concentration of 2.0 mg/mL was tested for its ability to block HCV entry into PBMCs (lane 10). Amplified products were resolved in 3% agarose gel, followed by ethidium bromide staining.

Camel casein without α-lactalbumin could not block HCV cell entry. One set of cells, PBMCs (2.5 × 105) or HepG2 cells (105), was cultured in duplicate, as described under Materials and Methods. One of the cultures was treated with 2.0 mg/mL casein for 60 min and then infected with HCV for 90 min. Camel casein could not protect the cells from HCV entry (Figure 2). The other cultures were inoculated with HCV-infected sera pretreated with casein (2.0 mg/mL) for 60 min. The inoculated cells were cultured for 8 days. In these cells, RT-nested PCR amplified a 174 bp region at the 5′ end of the HCV non-coding sequence in comparison to the positive control (cells infected with HCV) and negative controls (cells without infection). The 174-bp band was not detected in case of HepG2 cells, while it was detected in the case of PBMCs (Figure 2).

### 4.3. Effect of Camel Casein on Intracellular Replication of HCV

Camel casein with and without α-lactalbumin at concentrations of 0.25, 0.5, 0.75, 1.0, and 2.0 mg/mL was investigated for its ability to inhibit viral replication inside infected PBMC and HepG2 cells in vitro (data not shown).Inhibition of viral replication was detected by amplifying viral RNA segments, using RT-PCR. Casein could not inhibit HCV replication at concentrations of 0.25, 0.5, 0.75, 1.0, and 2.0 mg/mL after both first (4 days) and second doses (8 days) of treatment in both cell types. In case of casein with α-lactalbumin, the 174-bp band was not detected in HepG2 cells in case of casein with α-lactalbumin, while it was detected in PBMCs (data not shown).The differential appearance of the 174-bp band may be due to the reduced viability in HepG2 cells only.

### 4.4. Assay of HCV Immunostaining in PBMC and HepG2 Cells

**Figure 3:**
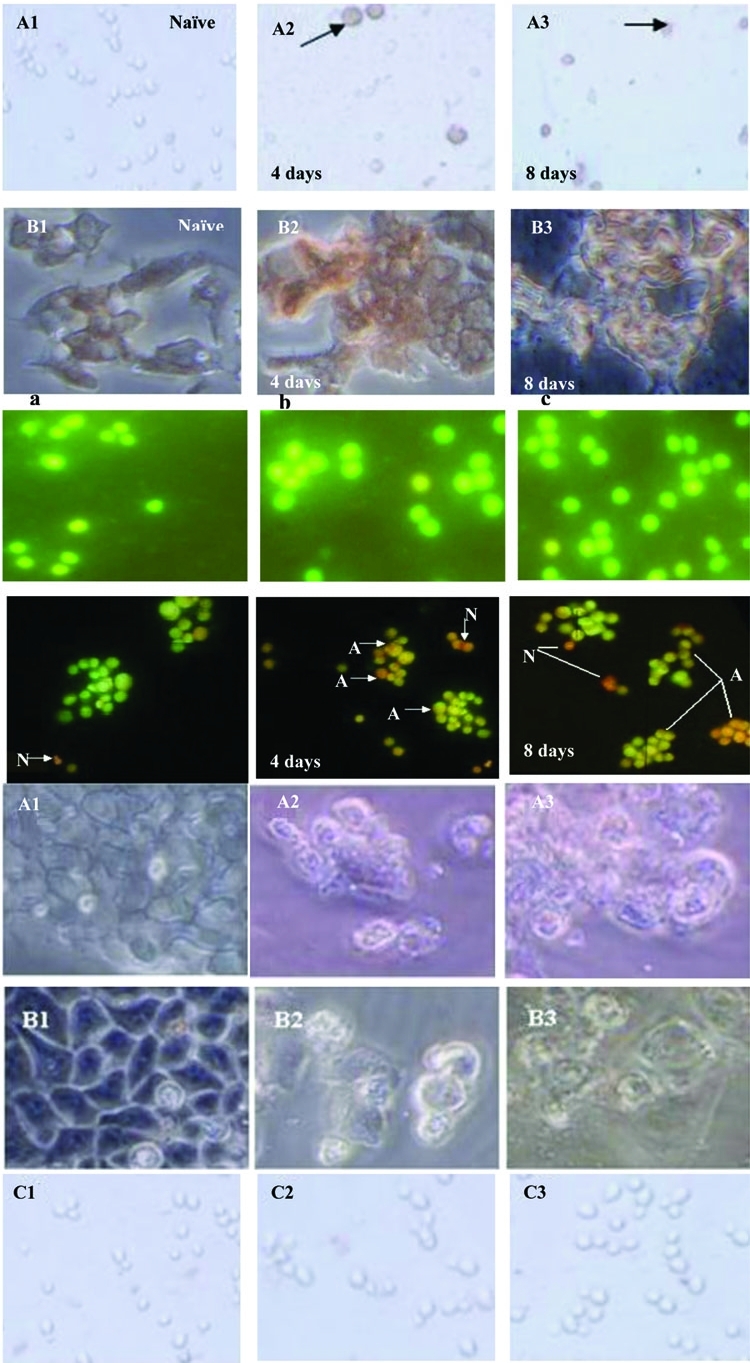
(I) Typical Pictures of HCV-Infected (A) PBMCs and (B) HepG2 cells under a light microscope (40x). Pictures A1 and B1 are the negative control. Pictures A2 and B2 are HCV-infected cells treated with camel casein for 4 days. Pictures A3 and B3 are HCV-infected cells treated with camel casein for 8 days. The arrows indicate the foci. (II) A panel indicates naïve human PBMCs and cells treated with casein (containing α-lactalbumin) at concentrations of 0.5 and 2.0 mg/mL (a, b, and c, respectively) for 8 days. Both naïve and treated cells were healthy. The panel below demonstrates the symptoms of apoptosis in HepG2 cells, which were visualized using the EB/AO method. Untreated HepG2 cells (a), and HepG2 cells were treated with 2.0 mg/mL camel casein for 4 days (b) and 8 days (c). (N) indicates necrotic cells; (A) indicates bright orange apoptotic cells. Normal green cells indicate non-apoptotic cells. (III) A panel showing the pictures of (A1-3) HepG2 cells, (B1-3) HeLa cells, and (C1-3) PBMCs under a light microscope (×20). Pictures A1, B1, and C1 show untreated cells. Pictures A2, B2, and C2 show cells treated with 0.5 mg/mL camel casein, and pictures A3, B3, and C3 show cells treated with 2.0 mg/mL casein. Camel casein did not exert any effect on PBMCs but exhibited typical apoptotic activity against HepG2 and HeLa cells.

Immunostaining assays have been widely used to evaluate the neutralizing antibody responses to viruses that can form foci in infected cells. After 8 days of infection and cell permeabilization, detection of the HCV foci was carried out using primary antibody and a peroxidase anti-human IgG probe. The reaction was developed with 3, 3′ diaminobenzidine (DAB) horseradish peroxidase substrate. The viral foci were stained brown, rendering them easily visualized under the light microscope. Figure 3 I demonstrates that the viral foci were clearly detected in PBMCs, while in HepG2 cells, the cell membrane did not appear to be firm, which might indicate apoptosis.

### 4.5. Cytotoxic Effect of Camel Casein

**Figure 4:**
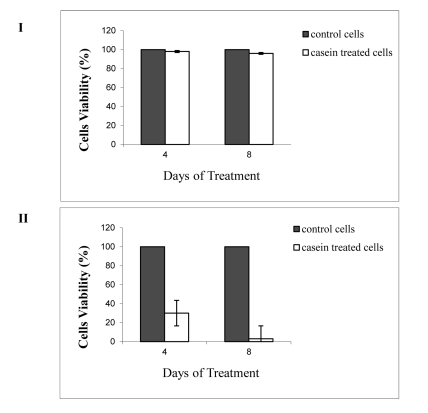
Viability of PBMCs (I) and HepG2 Cells (II) Treated with Camel Casein Containing α-Lactalbumin for 4 and 8 Days. Cell Viability was Examined by the MTT Assay, and the Data Presented are the mean ± standard Deviation of the Assays Conducted in Triplicate.

To rule out the possibility that the elimination of the HCV was caused by the reduced viability of PBMCs or HepG2 cells, the cytotoxic effects of camel casein (with α-lactalbumin) on the cells were examined. PBMCs (104) and HepG2 cells (104) were treated with camel casein (2 mg/mL) for 4 and 8 days, respectively. Cell viability was compared with that of untreated PBMCs and HepG2 cells. Casein had no adverse effects on the viability of PBMCs after incubation for 4 and 8 days. In the case of HepG2 cells, viability was reduced to 30% after 4 days of incubation and to 3% after 8 days of incubation (Figure 4). Microscopic examination revealed instability in both HepG2 and HeLa cell membranes, ranging from an unorganized to a destructive appearance (Figure 3). In contrast, casein (without α-lactalbumin) did not exert toxic effects on both cell lines (data not shown).

### 4.6. Camel Casein Induces Apoptosis in HepG2 and HeLa Cell Lines

The cytotoxic effect of casein containing α-lactalbumin on the cancer cell lines prompted us to examine casein’s apoptotic effect in comparison with its effect without α-lactalbumin. To check the ability of purified camel casein to initiate the apoptosis cascade in HepG2 and HeLa cells and PBMC cells, 0.5 and 2.0 mg/mL of purified casein were incubated with these cells for 4 and 8 days. We screened for apoptotic events using DNA fragmentation (DNA leader) and EB/AO staining and microscopic examination. Typical DNA laddering was clearly induced in HepG2 and HeLa cells (Figures 3 I, Figures 3 II, and Figures 3 III) at both concentrations of casein used (results for 0.5 mg are presented) but was absent in treated PBMC and untreated HepG2 cells. However, EB/AO staining results indicated DNA fragmentation. By using these methods, we determined the live and apoptotic cells in both suspension cells (PBMCs) and adherent cells (HepG2 and HeLa). The results indicated that casein had no effect on PBMC cells (Figure 4 I) at concentrations of 0.5 and 2.0 mg/mL, while it induced apoptosis in HepG2 and HeLa cells (Figure 4 II). Live cells showed normal green nuclei; early apoptotic cells exhibited bright green nuclei with condensed or fragmented chromatin; and late apoptotic cells displayed condensed and fragmented orange chromatin. A comparison of the effect of camel casein at concentrations 0.5 and 2.0 mg/mL on untreated and treated PBMCs, HepG2, and HeLa cells is presented in Figure 4 II.

## 5. Discussion

Numerous studies have shown that HCV genotype-4 patients have a response rate to interferon-α (IFN-α) monotherapy or combination therapy with ribavirin (RBV) that is less favorable than that of genotypes 2 and 3; and HCV genotype-4 patients have a response failure rate of approximately 60%. Resistance to antiviral therapy remains a serious problem in the management of chronic hepatitis C; thus, there is an urgent need for a novel interferon or drugs [4][15][16][17]. Several types of traditional medicines have been used by HCV patients in Egypt as supportive and/or adjuvant treatment, with camel milk being the most popular among these. Our previous examination of 3 camel milk proteins, namely, lactoferrin,amylase, and immunoglobulin G (IgGs), revealed that they had anti-HCV activity ranging from undetectable (amylase) to intermediate (IgGs) to strong (lactoferrin) [5][6][7][11]. In this study, we investigated the potential activity of camel casein against hepatitis C virus infectivity in HepG2 and PBMCs. Results show that camel casein failed to inhibit HCV entry into PBMCs and HepG2 cells, either by direct interaction with virus molecules or interaction with cells. HCV was eliminated by the reduced viability of HepG2 cells because camel casein induced apoptosis in and reduced the viability of HepG2 tumor cells. Consequently, HCV RNA was not detected when casein was used as the treatment, regardless of the casein concentration.Casein represents the main protein in milk. It is not present in milk as a single element; instead, it forms a large complex with calcium phosphate [18]. Casein consists of acidic proteins with pIs ranging between pH 4.9 and 5.9 and molecular weights ranging between 19 and 28 kDa. Although hitherto no biological activity has been ascertained for casein, it is nevertheless considered an important nutritional component, i.e., a source of bioactive peptides. No antiviral activity has been ascribed to intact casein [19], as in the current work. However, chemically modified caseins may acquire antiviral properties. In fact, a large number of bioactive peptides derived from the proteolytic hydrolysis of casein have been isolated and characterized. These peptides perform several biological activities such as antimicrobial [20], opioid agonistic and antagonistic [21], immunomodulatory [22], mineral-binding [23], antihypertensive [24] and antithrombotic [25] activities.The unexpected results we obtained were due to the cytotoxic effects of casein. The above mentioned results indicate that the α-lactalbumin in purified camel casein may represent the factor responsible for the current apoptosis induction in both HepG2 and HeLa cell lines. The present results are in agreement with those of Svensson et al. [26] who reported that upon unfolding, human and bovine α-lactalbumin can form a tumoricidal complex with oleic acid called human alpha-lactalbumin, which is lethal for tumor cells (HAMLET). This complex induces differential and significant apoptosis in several cancer lines [14][27][28]. Camel casein was not able to block or neutralize HCV cell entry either by direct or indirect interaction. However, casein could induce the apoptosis cascade in both HepG2 and HeLa cells.
